# Development and Evaluation of Sedentary Time Cut-Points for the activPAL in Adults Using the GGIR R-Package

**DOI:** 10.3390/ijerph20032293

**Published:** 2023-01-27

**Authors:** Duncan S. Buchan, Julien S. Baker

**Affiliations:** 1 Division of Sport and Exercise, School of Health and Life Sciences, University of the West of Scotland, Lanarkshire Campus, Glasgow G72 0LH, Scotland, UK; 2 Research Centre for Population Health and Medical Informatics, Hong Kong Baptist University, Kowloon Tong, Hong Kong, China

**Keywords:** agreement, auto-calibration, equivalence, free-living, criterion validity, accelerometry

## Abstract

The purpose of this study was to develop sedentary cut-points for the activPAL and evaluate their performance against a criterion measure (i.e., activPAL processed by PALbatch). Part 1: Thirty-five adults (23.4 ± 3.6 years) completed 12 laboratory activities (6 sedentary and 6 non-sedentary activities). Receiver operator characteristic (ROC) curves proposed optimal Euclidean Norm Minus One (ENMO) and Mean Amplitude Deviation (MAD) cut-points of 26.4 mg (ENMO) and 30.1 mg (MAD). Part 2: Thirty-eight adults (22.6 ± 4.1 years) wore an activPAL during free-living. Estimates from PALbatch and MAD revealed a mean percent error (MPE) of 2.2%, mean absolute percent error (MAPE) of 6.5%, limits of agreement (LoA) of 19% with absolute and relative equivalence zones of 5% and 0.3 SD. Estimates from PALbatch and ENMO revealed an MPE of −10.6%, MAPE of 14.4%, LoA of 31% and 16% and 1 SD equivalence zones. After standing was isolated from sedentary behaviours, ROC analysis proposed an optimal cut-off of 21.9 mg (herein ENMOs). Estimates from PALbatch and ENMOs revealed an MPE of 3.1%, MAPE of 7.5%, LoA of 25% and 9% and 0.5 SD equivalence zones. The MAD and ENMOs cut-points performed best in discriminating between sedentary and non-sedentary activity during free-living.

## 1. Introduction

Sedentary behaviour (SB) is defined as any waking behaviour characterized by an energy expenditure ≤1.5 metabolic equivalents, while in a sitting, reclining or lying position [1]. The health consequences of excessive sedentary time are well established with recent meta-analyses reporting a non-linear positive dose-response relationship for time spent sedentary with all-cause mortality and cardiovascular disease (CVD) mortality [2,3]. Recent estimates from studies that have captured time spent sedentary using accelerometers, indicate that adults spend approximately 8 h/day sedentary [4]. These estimates are broadly in line with recently proposed thresholds of 6–8 h/day of total sitting time whereby the risk for all-cause and CVD mortality increases rapidly [2] and ≥9.5 h/day of sedentary time for higher risk of death [3]. From these findings it seems clear that substantial health benefits can be gained by limiting the time individuals are sedentary and replacing this time with more physical activity (PA). Therefore, being able to correctly identify SB and separate it from light-intensity physical activity (LPA) is crucial. Doing so, would enhance our understanding of the relationships between SB and health indicators as well as the health improvements that may be seen if intervening to reduce SB.

The gold standard device for the objective measurement of SB is the thigh-worn activPAL (PAL Technologies Ltd., Glasgow, UK) [5]. The activPAL device has demonstrated a sensitivity of between 96% to 98% for correctly identifying SB against direct observation in laboratory based studies replicating activities of daily living [6,7]. Precise estimates of SB were also evident under free-livings conditions that consisted of two 6 h observations, as well as demonstrating a sensitivity to reductions in sitting time [8]. Although the activPAL proprietary software has a built in algorithm that can estimate energy expenditure (expressed as metabolic equivalents (METs)) [9], time spent in moderate-vigorous PA (MVPA) is not usually provided. Previous studies have found the activPAL to overestimate METs at slower walking speeds but underestimate METs at faster walking speeds when compared against indirect calorimetry [10,11]. Furthermore, the activPAL has been shown to overestimate time spent in MVPA compared to the ActiGraph (ActiGraph, Pensacola, FL) when worn concurrently [12]. As many research-grade accelerometers (i.e., ActiGraph, Axivity and GENEActiv) are unable to differentiate between postures, researchers interested in capturing both PA and SB require study participants to wear ActiGraph and activPAL devices concurrently [13,14]. Clearly such an approach would provide valuable insights into PA and SB given their prominence within recent international PA guidelines [15,16,17]. Nonetheless, such approaches are not cost effective and can increase the burden for research participants.

With recent technological advancements, tri-axial research grade accelerometers can provide users with the collected raw accelerometer data that can facilitate comparisons between devices using identical processing methods. Using open-source accelerometer processing and analyzing software such as GGIR, previous studies have examined the comparability of the same/different devices within and between body locations with promising findings for future data harmonization [18,19,20]. The widely used raw acceleration MVPA cut-point of 100 milli-gravitational units (mg) is often applied to raw acceleration accelerometer data to estimate time spent in MVPA, and to facilitate comparisons between devices [18,21]. Laboratory derived cut-points have also been proposed for adults to estimate time spent sedentary [22,23,24], yet subsequent cross-validation of these in free-living settings against a thigh-worn criterion method (i.e., activPAL or axivity) tend to show modest accuracy [22,25]. Several factors may influence these findings including differences in device wear locations, sampling frequencies, processing methods, algorithms to detect non-wear as well as the limited number of activities used in the laboratory protocol by Hildebrand and colleagues. Future research should aim to address these issues, where possible, to minimize the influence of factors which may exacerbate differences in the time spent sedentary.

One such approach that removes the reliance upon using proprietary algorithms from PAL Technologies Ltd. to process data collected from the activPAL device has recently been proposed [20]. As the activPAL device collects raw acceleration data across three axis, the raw data can be downloaded using PAL Technologies Ltd. freely available software and saved in raw format as .csv files, to be subsequently processed using the open-source software GGIR [20,26]. Notwithstanding the obvious benefits of transparency and reproducibility for the research community when using GGIR, users also have the ability to adapt and expand the functionality of GGIR by specifying certain input arguments and/or selecting certain output variables. When using the raw acceleration data for instance, users can quantify the overall levels of activity, the intensity distribution across the monitoring period, as well as describing the intensity of the most active periods of the day across a user defined duration. The potential therefore of reporting these outcomes alongside validated raw acceleration cut-points that can quantify the time spent sedentary, holds enormous appeal. Yet to the best of our knowledge, no raw acceleration cut-points have been proposed that can quanitify time spent sedentary for the activPAL device. In view of the gaps in the literature identified above, the aims of this study were: (1) To provide activPAL specific cut-points for discriminating between SB and typical light-intensity physical activities using the open-source software GGIR (part 1); (2) To explore the performance of the cut-points in an independent sample during free-living (part 2).

## 2. Materials and Methods

### 2.1. Laboratory-Based

A convenience sample of thirty-five adults (14 females; age 23.4 ± 3.6 years; BMI = 23.6 ± 3.1 kg/m^2^) were recruited from the University of the West of Scotland student body via email and word of mouth. All participants were informed of the study aims and provided informed consent, after approval from the ethics committee of the University of the West of Scotland (application 8692-7016). Data collection took place between September 2019 and November 2019.

#### 2.1.1. Procedures

All study procedures were explained to participants upon arrival at the laboratory. Thereafter, participants were asked to wear an activPAL Micro4 (PAL Technologies LTD, Glasgow, UK; herein activPAL) on the anterior midline of the right thigh using nitrile sleeves and a Hypafix dressing. The activPAL is a triaxial accelerometer with a dynamic range of ±4 g. ActivPAL devices were initialized using PAL Connect version v8.10.8.75 to record data using the default settings (20 Hz, 10 s minimum sitting and upright period). The same computer was used to initialize all devices which were programmed to commence data collection after distribution.

Once fitted with the activPAL, participants performed 12 activities in a sequential order which included 4 lying positions, 2 sitting positions and 6 upright positions (See Table 1 for a description of the activities). In the main, each activity lasted for 5 min, separated by a 30 s break. Whereas activity 12 lasted for 2 min with a break of 2 min provided between activities 11 and 12. The start and end times of each activity was recorded for each participant using a digital watch synchronized with the computer which initialized the activPAL devices. All participants were observed by the research team whilst completing the activities which lasted approximately 70 min.

#### 2.1.2. Data Reduction and Processing

Data was downloaded using PAL batch v8.11.1.63 and saved in raw format as time-stamped .csv files. These .csv files were then processed using the GGIR package v2.6-0 in R statistical software (R Foundation for Statistical Computing, Vienna, Austria, https://cran.r-project.org/, accessed on 15 January 2023) [26]. GGIR detected sustained and abnormally high values, non-wear time and computed the Euclidean Norm Minus One (ENMO), with negative values rounded up to zero, and Mean Amplitude Deviation (MAD) metrics. Since ENMO is sensitive to poor calibration [27], back-up calibration coefficients provided from the same activPAL device worn during free-living was used in GGIR as described previously [21,22]. This was necessary due to the short duration of the laboratory protocol and the absence of sufficient periods of non-movement which is needed for auto-calibration in GGIR. These files were subsequently exported to Microsoft Excel v16.61.1 (Microsoft, Redmond, WA, USA) for analysis using a macro developed for the laboratory protocol data. When applying the macro, the first and last 30 s of data from each activity were excluded to provide average values for MAD and ENMO for each activity, averaged over 5 s epochs, and expressed in milli-gravitational units (mg). Participant files were used in subsequent analysis if post-calibration error was <0.02 g.

### 2.2. Free-Living

As part of a separate study, 38 adults (15 females; age 22.6 ± 4.1 years; BMI = 22.4 ± 3.5 kg/m^2^) were recruited from the University of the West of Scotland student body via email and word of mouth and instructed to wear the activPAL device for 8 consecutive days during free-living. Ethical approval for the study was received from the University of the West of Scotland with data collection taking place between October 2019 to December 2019 (application 16818-14107). All participants were informed of the study aims and provided written informed consent.

#### 2.2.1. Procedures

Participants were asked to wear an activPAL device on the anterior midline of the right thigh using nitrile sleeves and a Hypafix dressing to waterproof [28]. ActivPAL devices were initialized using PAL Connect version v8.10.8.75 to record data using the default settings (20 Hz, 10 s minimum sitting and upright period). The same computer was used to initialize all devices and programmed to commence data collection after distribution. Participants were fitted with the device prior to leaving the data collection session and requested to wear the device at all times for 8 days.

#### 2.2.2. Data Reduction and Processing

Upon return of the devices, data was downloaded using PAL batch v8.11.1.63 and saved in raw format as .csv files. These files were subsequently processed in GGIR package v2.7-2 in R statistical software (R Foundation for Statistical Computing, Vienna, Austria, https://cran.r-project.org/, accessed on 29 March 2022) which detected sustained and abnormally high values, non-wear time and auto calibrated the files using local gravity as a reference [27]. The GGIR package calculated both ENMO and MAD averaged over 5 s epochs, expressed in mg [29]. To enhance generalizability, non-wear was imputed using the default settings in GGIR whereby invalid data were imputed by the average at similar times of different days of the monitoring period. Participant files were used in subsequent analysis if post-calibration error was <0.02 g and participants had ≥1 day of valid wear data (defined as 24 h per day). The participant files that met the inclusion criteria after being processed in GGIR, also had to provide ≥1 day of valid wear data (defined as 24 h per day) when processed in PAL batch v8.10.12.60. Thus, data files for each day provided by GGIR and PAL Batch were visually inspected to ensure outcomes were compared using identical timeframes and days. Furthermore, as one of the aims of this study was to evaluate the performance of laboratory-based cut-points in a free-living setting, sleep data was excluded from subsequent analysis. To facilitate this, the start and end of the time in bed provided by PAL batch was used to estimate sleep time for each valid day. A sleep log was subsequently created for all participants using the start and end time in bed provided by PAL batch, in GGIR. This ensured that the sleep estimates were the same between both processing methods (i.e., PAL batch and GGIR) and helped minimize bias when comparing outcomes between the two processing methods. Finally, time spent in SB provided by PAL batch was used as the criterion measure in subsequent analysis.

#### 2.2.3. Statistical Analysis

All lying down and sitting activities (activities 1–6) were grouped together and considered as sedentary behaviours. Receiver operating characteristic (ROC) analyses were then undertaken to identify optimum ENMO and MAD cut-points to distinguish between sedentary and non-sedentary behaviours (i.e., activities 7–12). In the ROC analyses, the Youden index was used, defined as Youden = sensitivity + specificity −1, to optimize sensitivity and specificity and to determine the optimal MAD and ENMO cut-points [30]. To interpret the accuracy of the cut-points, the area under the curve (AUC) was provided for each cut-point with values < 0.7, 0.70–0.79, 0.80–0.89 and ≥0.90 considered poor, fair, good and excellent, respectively [31]. Prior to undertaking the ROC analyses, events files provided by PAL batch were downloaded and visually inspected to confirm the correct posture (i.e., sedentary, non-sedentary) was identified.

Using the free-living data (part 2), time spent in SB was provided by PAL batch for each participant that met the inclusion criteria. Thereafter, the optimal cut-points for MAD and ENMO provided by the ROC analyses were applied in GGIR to estimate time spent below these thresholds (herein termed sedentary time). Agreement between time spent in SB from PAL batch and sedentary time from GGIR was examined using mean percent error (MPE), mean absolute percent error (MAPE), equivalence tests and Bland-Altman plots as recommended [32]. As reported previously, a 5% threshold was used to aid the interpretation the MPE findings and consider the practical relevance of the generated cut-points [33]. Pairwise 95% equivalence tests were used to establish whether the 95%CI of the mean for sedentary time fell within the proposed equivalence zone for SB [34]. Rather than state a fixed absolute zone to infer equivalence, the required percentage needed to reach equivalence is provided alongside the zone necessary to achieve equivalence as a proportion of the SD [35]. Finally, Bland-Altman plots were used to assess agreement between each processing method and to visualize the magnitude of any differences [36]. Statistical analyses were undertaken using IBM SPSS statistical software for Windows version 25 (IBM, Armonk, NY, USA). Descriptive statistics were calculated for all outcomes (mean ± SD) or median (25th–75th percentile) following normality testing. ROC curve analyses were undertaken using MedCalc 14.8.1 (MedCalc Software, Flanders, Belgium) whereas equivalence testing was undertaken in Minitab (v17) with alpha set at 0.05.

## 3. Results

### 3.1. Laboratory Based

The thirty-five participants completed all activities with their data files meeting the inclusion criteria. The ENMO and MAD values for the sedentary and non-sedentary behaviours are provided in Table 2. The ENMO values tended to be higher for sedentary behaviours and standing compared to MAD, whereas MAD values tended to be higher for the self-paced walk and ascending/descending stairs. Findings from the ROC analyses revealed excellent classification accuracy for both ENMO and MAD models, with AUC values of 1. For ENMO, an acceleration value of 26.4 mg and 30.1 mg for MAD was found to discriminate sedentary vs. non-sedentary behaviours.

### 3.2. Free-Living

Of the thirty-eight participants recruited, 2 failed to provide 24 h of wear time for ≥1 day (confirmed by GGIR and PAL batch) and were removed from subsequent analysis. This left thirty-six participant data files (14 females; age = 28.5 ± 3.6 years; BMI = 24.5 ± 3.1 kg/m^2^) to be examined in subsequent analysis. No post-calibration error > 0.01 g was evident from these data files when processed through GGIR. The activPAL was worn on average for 5.7 ± 1.5 days with outcomes from 205 days available for analysis. Estimates of time spent sedentary from PAL batch was 548.8 ± 85 min/d and sedentary time from GGIR were 613.8 ± 116.1 min/d for the ENMO metric and 561.3 ± 92.3 min/d for the MAD metric. Findings from the MPE and MAPE analyses can be found in Table 3. Given the poorer performance of the ENMO cut-point compared to MAD, standing still (passive standing [1]) was isolated from SB in the laboratory and the optimal ENMO cut-point was provided by the ROC analysis to separate SB vs. passive standing. An ENMO acceleration value of 21.9 mg (herein ENMOs) was found to discriminate SB vs. passive standing. Despite the poor classification accuracy (AUC = 0.58), sensitivity was near perfect (97%) with specificity lower at 43% for the ENMOs cut-point. Time spent sedentary for ENMOs was 574.7 ± 121.3 min/d. Performance of the ENMOs cut-point was then examined in subsequent analysis.

**Figure 1:**
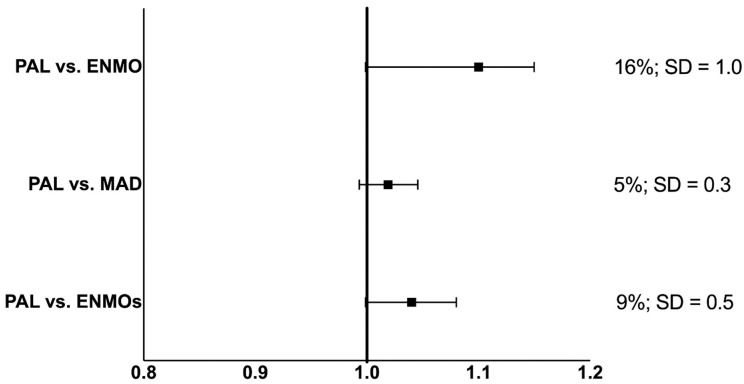
Equivalence between estimates of time spent sedentary between different processing methods. To the right of the figure, the absolute zone needed to reach equivalence is provided as a %, alongside the zone necessary to achieve equivalence as a proportion of the SD. The PALbatch software was used as the reference in all analyses.

The lowest MPE evident between processing methods was between PAL batch and MAD at −2.2% with the highest MPE evident between PAL batch and ENMO at −10.6%. Individual level differences followed a similar trend with the lowest MAPE evident between PAL batch and MAD at 6.5%, whereas the highest MAPE was evident between PAL batch and ENMO at 14.4%. Findings from the equivalence analyses are provided in Figure 1. The absolute zone needed to reach equivalence for time spent sedentary provided by PAL batch and ENMO was 16%. This corresponded to a relative zone of 1 SD to reach equivalence. Comparisons between PAL batch and MAD revealed an absolute zone of 5% which corresponded to a relative zone of 0.3 SD to reach equivalence. When comparing sedentary estimates from PAL batch and ENMOs, the absolute zone needed to reach equivalence was 9% which corresponded to a relative zone of 0.5 SD.

**Figure 2:**
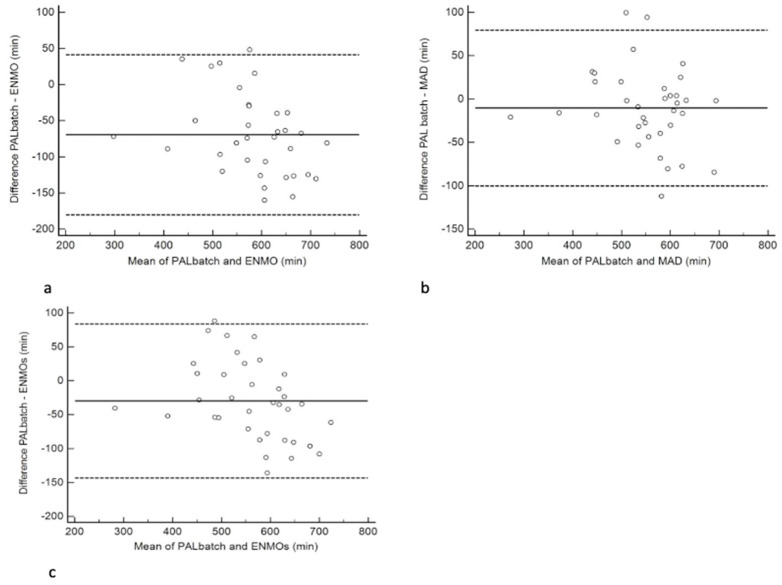
Bland-Altman plots evaluating the agreement between estimates of time spent sedentary between different processing methods. Mean bias is represented by a solid line; 95% limits of agreement with dashed lines. (**a**) Estimates of sedentary behaviour from PALbatch vs. ENMO from GGIR, (**b**) Estimates of sedentary behaviour from PALbatch vs. MAD from GGIR, and (**c**) Estimates of sedentary behaviour from PALbatch vs. ENMOs from GGIR.

From the Bland-Altman analyses provided in Figure 2a–c, mean bias between PAL batch and ENMO was −70 min with limits of agreement (LoA) of—180 min to 41 min. This equated to a mean bias of −12% and LoA of ±31%. The mean bias between PAL batch and MAD was −11 min with LoA of −100 min to 79 min. This equated to a mean bias of −2% and LoA of ±19%. The mean bias between PAL batch and ENMOs was −30 min with LoA of −143 min to 84 min. This equated to a mean bias of −5% and LoA of ±25%.

## 4. Discussion

This is the first study to develop ENMO and MAD cut-points for the activPAL when worn on the thigh to estimate time spent sedentary. Moreover, the performance of these cut-points were subsequently evaluated in an independent sample during free-living. The ENMO and MAD cut-points generated from the ROC analysis demonstrated excellent discrimination between sedentary and non-sedentary behaviours. This suggested that adults who are sedentary have ENMO and MAD values below the generated cut-points and are unlikely to be classified as being physically active. When applying the cut-points to free-living data, the MAD cut-points performed best demonstrating good levels of agreement (MPE = −2.2%) and equivalence (5%; ≤0.3 SD) with SB values from PAL batch. After isolating standing still from SB, the developed ENMOs cut-point was applied to free-living data and demonstrated good levels of agreement (MPE = 3.1%), equivalence (9%; 0.5 SD) and a smaller confidence interval from the Bland-Altman plot compared to ENMO. These findings suggest that the MAD cut-point of 30 mg can be used to discriminate between sedentary and non-sedentary behaviours, whereas the ENMOs cut-point of 22 mg can be used to discriminate between SB and standing. Applying these cut-points to free-living data demonstrated comparable sedentary estimates to that of the gold standard device for the objective measurement of SB.

It is not possible to draw comparisons of the developed cut-points with those previously published since no other study has reported cut-points for the activPAL device using the processing methods detailed here. Nonetheless, comparisons can be made with other studies that have looked to propose sedentary cut-points from other accelerometers worn on different wear sites. Findings from this study revealed that the magnitude of accelerations were considerably larger for activities that required participants to move whilst standing, as observed elsewhere [24,37]. For instance, in the study by Sanders et al., [24] adults aged ≥ 60 years wore a GENEActiv device on their non-dominant wrist and an ActiGraph device on their left hip whilst completing sixteen structured activities in a laboratory. When comparing the average ENMO values between household chores (i.e., washing up at a sink and mopping the floor) and sitting from both the GENEActiv (128 mg vs. 8 mg) and the ActiGraph (15 mg vs. 3 mg), the magnitude of accelerations between these activities were evident. Larger differences were also evident when comparing ENMO values when walking on a treadmill (GENEActiv = 209 mg; ActiGraph = 105 mg) to that of sitting [24].

In a similar study, young adults were asked to wear an ActiGraph and GENEActiv device on their right hip, and the same devices on their non-dominant wrist whilst performing 16 activities in a laboratory setting [37]. In this study, both MAD and ENMO acceleration values are provided for the activities undertaken. A clear distinction in acceleration values was evident between sedentary behaviours and light intensity activities requiring ambulation, regardless of metric or device location. For instance, average ENMO acceleration values for sedentary behaviours from the wrist and hip were approximately 10 mg and 5 mg, respectively, after averaging values from both accelerometer devices. Similar values were evident for MAD from the wrist and hip. When examining the acceleration values for a self-paced free-living walk, average values tended to fall between 50 mg to 150 mg regardless of device, metric, or location. In this study, the average acceleration values associated with the self-paced walking activity were larger (∼270 mg for ENMO and ∼331 mg for MAD) than those reported from younger [37] and older adults who walked on a treadmill [24].

Differences in acceleration values between laboratory-based validation studies are to be expected, even if the same or similar activities are undertaken across studies. Much like the generation of accelerometer cut-points from laboratory validation studies, the cut-points, or acceleration values, are population and protocol specific. Moreover, differences in acceleration values from devices worn on the thigh to devices worn on the hip and wrist also reflect the different movements at each location i.e., wrist movements can be independent of body posture and ambulation. Although attempts were made in this study to design lab-based activities that reflect free-living activities, it is possible that such attempts may not sufficiently capture the typical movements in free-living environments. Therefore, and in accordance with best practice recommendations [38], performance of the generated cut-points were evaluated in an independent sample during free-living against a criterion measure (i.e., activPAL). The MAD cut-point of 30 mg performed best followed by the ENMOs cut-point of 22 mg, despite its poor classification accuracy. The poor classification accuracy of the ENMOs cut point is likely a consequence of the similar acceleration values evident between the sedentary behaviours and the standing activity. As the sensitivity of the ENMOs cut-point was near perfect however, there is little risk of individuals being misclassified as being physically active as was found during free-living. Furthermore, these findings highlight the importance of evaluating cut-points that are generated in a simulated laboratory environment within a free-living setting.

From the previous validation studies that generated ENMO and MAD SB cut-points for adults [22,24,37], only Hildebrand et al. [22] evaluated the performance of the generated cut-points in a free-living setting against a criterion measure (activPAL). The authors evaluated the performance of their developed cut-points for the non-dominant wrist and hip during free-living by comparing the percentage of time correctly identified as sedentary (sensitivity) and non-sedentary (specificity) against the activPAL. Sedentary time estimates were found to be significantly higher compared to the activPAL regardless of the accelerometer device (ActiGraph or GENEActiv) or wear site with differences ranging from 84% to 86% from the hip and 69% to 72% from the wrist. When reviewing the absolute agreement findings across all devices and locations, specificity was poor ranging from 26% to 49% regardless of device and location. This suggests that non sedentary behaviours that were undertaken with minimal ambulation were likely incorrectly classified as sedentary behaviour and may explain the large mean differences in sedentary time estimates reported by the authors. Moreover, sedentary estimates were compared between different body locations and in the case of the non-dominant wrist and thigh, movements could be independent on one another which could also explain the findings of this study. Nonetheless, the mean differences in sedentary estimates during free-living when applying the cut-points used in this study were considerably less than those reported by Hildebrand et al. [22], providing confidence in the proposed cut-points.

The acceleration values of the proposed ENMO and MAD cut-points are similar in magnitude, but there are differences between these metrics that limits their comparability [29]. As the raw acceleration signal contains both the movement and gravitational components, these need to be separated. The ENMO metric removes the gravitational component by subtracting one gravitational unit from the Euclidean Norm of the three raw acceleration signals, to provide the movement component of the acceleration signal (i.e., ENMO) [37]. Whereas for the MAD metric, gravity is estimated as the average acceleration per moving time window. The problem with this approach however, is that the moving average of the acceleration signal may reflect gravitational acceleration as well as low frequency movements [29]. When the gravitational and movement components are then separated by the GGIR algorithm to provide the MAD metric, lower amplitude movements may also be removed by the filter. When you consider the differences in ENMO and MAD values for the sedentary behaviours and standing activity observed in this study (Table 2), the higher ENMO values may be a consequence of the different methods used to separate the raw acceleration signal. Support for this assumption can be seen from a recent study which compared the ENMO and MAD values provided from ActiGraph devices when worn at the hip and both wrists [39]. The authors reported that agreement between ENMO and MAD was lower during sleeping hours for all wear sites. This is likely a consequence of the lower magnitude of acceleration values evident during this time period which resulted in lower mean values for MAD compared to ENMO, across all wear sites. In contrast, higher mean acceleration values were evident for MAD compared to ENMO during waking hours. Although in this study the sleep period was removed from subsequent analyses, the findings from Migueles et al., [39] and in this study suggest that sedentary time comparisons between the MAD and ENMO cut-points should be done with caution.

With the activPAL considered the gold standard device for the measurement of SB, the cut-points proposed in this study should not be considered as a replacement for the PAL analysis software given the wealth of SB related outcomes provided. Rather, these cut-points provide an additional means for researchers to analyze and interpret their accelerometer data and explore associations with health outcomes alongside other outcomes provided by GGIR. When using GGIR, researchers are able to report on several additional outcomes (i.e., average acceleration; intensity gradient; MX and time when the most/least X h of activity is undertaken), other gravitational metrics (i.e., ENMO; MAD etc.) as well as the user having the ability to specify their own intensity-related thresholds and data reduction approaches [40]. Therefore, the cut-points obtained in this study can be used to provide a simple means of estimating time spent sedentary that is comparable to estimates provided by a criterion measure. Moreover, researchers can have confidence in these laboratory derived cut-points due to their performance in an independent sample during free-living across an 8-day monitoring period.

This study has several strengths including being the first to develop ENMO and MAD cut-points for the activPAL using the open-source software GGIR. The laboratory protocol consisted of 12 activities that were included to mimic the activities and movements undertaken by adults in a free-living setting. Thereafter, the performance of the developed cut-points was evaluated in an independent sample during free-living across 8 days. Another strength of the study is the use of the same procedures to identify, and remove, sleeping hours from subsequent comparisons. Moreover, using complete 24 h data when comparing outcomes removed the need for different algorithms to detect non-wear. The free-living participants demonstrated high compliance which strengthens the ecological validity of the accelerometer data. Finally, the ENMO metric is sensitive to poor calibration [29]. Therefore, autocalibration was undertaken for all accelerometer files used in this study. Limitations include the homogenous populations used in this study which limits the generalizability of our findings. Furthermore, the limited number of activities undertaken in the laboratory may also be seen as a limitation.

## 5. Conclusions

In conclusion, the ENMOs and MAD cut-points developed in the laboratory performed well when applied to an independent population during free-living and supports their practical relevance. Estimates of time spent sedentary were comparable to estimates provided by a criterion measure, with the MAD cut-point performing best in comparison to ENMOs. These findings suggest that users are able to process their collected activPAL data using GGIR and apply the ENMOs and MAD cut-point to estimate time spent sedentary alongside other GGIR metrics and outcomes. Future research may wish to undertake additional validation studies to propose MVPA cut-points from the activPAL device to be used alongside the cut-points proposed here.

## Figures and Tables

**Table 1:** Overview of the sedentary behaviours and light-intensity physical activities undertaken.

Posture	Activity	
Lying down	1	Lying on back with legs straight
	2	Lying on back with legs bent
	3	Lying on side with legs straight
	4	Lying on side with legs bent
Sitting	5	Sitting on a chair typing on a computer
	6	Sitting whilst texting on a mobile phone
Upright	7	Standing whilst using their mobile phone to browse the internet
	8	Self-paced walk in a forward direction around the laboratory
	9	Picking up items on the floor and placing them on a desk
	10	Dusting a set area
	11	Sweeping the floor of a set area
	† 12	Ascend then descend a flight of stairs (out with the laboratory)

† Activities lasted for 5 min, apart from activity 12 which lasted for 2 min.

**Table 2:** ENMO and MAD values for sedentary and non-sedentary behaviours.

Activity	ENMO (mg)	MAD (mg)
Sedentary behaviours	5.1 (3.1–8.6)	4.0 (1.8–9.1)
Standing whilst using their mobile phone to browse the internet	5.6 (3.6–31.4)	4.5 (1.9–6.2)
Self-paced walk in a forward direction around the laboratory	240.5 (209.4–341.7)	316.3 (268.7–370.2)
Picking up items on the floor and placing them on a desk	201.3 (174.5–214.3)	245.4 (210.4–259.9)
Dusting a set area	73.4 (47.4–87.7)	60.2 (50.7–71.3)
Sweeping the floor of a set area	94.4 (65.1–106.9)	77.1 (57.1–88.3)
Ascend then descend a flight of stairs (out with the laboratory)	248.7 (217.2–269.2)	349.7 (298.3–364.5)

Data are presented as median (25th–75th percentile). ENMO: Euclidean Norm Minus One (ENMO) measured in milligravity units (mg). MAD: Mean Amplitude Deviation (MAD) measured in milligravity units (mg).

**Table 3:** Agreement of time spent sedentary from the activPAL when processed using PAL batch and GGIR.

Criterion	Comparison	Mean ± SD Minutes	MPE ± SD	MAPE ± SD
PALbatch		548 ± 85.1		
	ENMO	613.8 ± 116.1	−10.6 ± 26.7	14.4 ± 12.1
	MAD	561.3 ± 92.3	−2.2 ± 7.9	6.5 ± 5.6
	ENMOs	574.7 ± 121.3	3.1 ± 11.4	7.5 ± 6.9

MAPE, Mean Absolute Percent Error; MPE, Mean Percent Error; ENMO, Euclidean Norm Minus One; MAD, Mean Amplitude Deviation ENMOs, Euclidean Norm Minus One cut-point separating sedentary behaviour vs. passive standing.

## Data Availability

The data presented in this study are available on reasonable request from the corresponding author.
